# Four years post-horsegate: an update of measures and actions put in place following the horsemeat incident of 2013

**DOI:** 10.1038/s41538-017-0007-z

**Published:** 2017-11-06

**Authors:** Stephanie Brooks, Christopher T. Elliott, Michelle Spence, Christine Walsh, Moira Dean

**Affiliations:** 1 Institute for Global Food Security, Queen’s University Belfast, Belfast, BT9 5BN Northern Ireland UK; 2 Agriculture and Horticulture Development Board Beef and Lamb, Stoneleigh Park, Warwickshire, CV8 2TL UK

**Keywords:** Agriculture, Business and industry

## Abstract

Complexities in food supply chains were highlighted by the so called ‘horsegate’ crisis in 2013, where beef meat was fraudulently adulterated with horse meat causing widespread recalls and subsequent investigations across both retail and food service markets in the European Union (EU). The beef supply chain is a complex supply chain, with global (EU and Non EU) sourcing strategies in order to secure supply. However, managing these complex supply chains can be difficult and consequentially can expose vulnerabilities similar to that of horsemeat, where horsemeat was found in beef meat within EU supply chains. Six months after the crisis broke, an independent review into the integrity and assurance of food supply networks was commissioned by the UK government and undertaken by Professor Chris Elliott of Queen’s University, Belfast. The review recommended eight pillars of food integrity to industry and government: consumers first, zero tolerance, intelligence gathering, laboratory services, audit, government support, leadership and crisis management. This article examines the extent to which these recommendations have been implemented using personal communications from Professor Chris Elliott and relevant industry bodies. Following the review, industry attitudes have changed substantially, testing and surveillance systems have been integrated into normal industry practice and the government is more prepared for future incidents through the establishment of the National Food Crime Unit (NFCU). Horsegate raised the profile of food fraud and crime in supply chains and despite improvements to date, further collaboration between industry and government is required in order to align fully with the recommendations.

## Food safety: Food fraud prevention is improved after the ‘horsegate’ scandal

Industry and government have learned their lessons after the incident in 2013 where beef meat was substituted with horsemeat. Using personal communications with representatives of relevant industry bodies, Moira Dean from the Queen’s University Belfast in UK and her colleagues examined measures the UK government and food industry have taken in response to the horsemeat scandal. They found significant improvement in the understanding and prevention of food fraud, with well-integrated testing and surveillance systems, along with a new government body to take a leadership role in such crises. Yet, collaboration between industry and government is yet to be enhanced. Given the complexities of international food supply chains, the UK lessons and recommendations to improve the integrity of food supply systems, set out in a review commissioned after the horsemeat crisis are relevant to other countries.

## Introduction

Several scandals/scares have shook the food industry, some deliberate and others accidental over the last four decades. These include, the Spanish cooking oil disaster of 1981, the Bovine Spongiform Encephalopathy outbreak of 1996, the 2011 German *Escherichia coli* O104:H4 outbreak in vegetation, the Irish pork dioxin crisis of 2008, and the 2008 Melamine crisis in infant formula in China, to name a few. More recently, the idea of food fraud or food crime has surfaced as a deliberate and sophisticated practice carried out by seasoned criminals on deceiving customers and/or consumers of the true nature of the product for financial gain.^1,2^ One of the latest and most high profile food crime-based scandals was that of horsemeat, or so called ‘horsegate’ in 2013.

## Background—‘horsegate’

In January 2013, following testing by the Food Safety Authority of Ireland as part of normal proactive monitoring activities, the horsemeat scandal broke. Horsemeat had been found in beef meat products sold in retail and food service markets throughout the United Kingdom (UK) and Ireland (https://www.fsai.ie/news_centre/press_releases/horseDNA15012013.html). Testing revealed beef products had been adulterated with horsemeat such that horse DNA was identified in 37% of beef burgers purchased from food retail stores including Tesco, Dunnes, Lidl and Aldi, all originating from three meat plants in the UK and Ireland (http://webarchive.nationalarchives.gov.uk/20150624093026/http://www.food.gov.uk/enforcement/monitoring/horse-meat/timeline-horsemeat). In February 2013, UK company Findus and retailers Aldi and Tesco reported finding horsemeat in their lasagne, spaghetti bolognese, burger and meatball products, all of which were produced by a French supplier (http://webarchive.nationalarchives.gov.uk/20150624093026/http://www.food.gov.uk/enforcement/monitoring/horse-meat/timeline-horsemeat).

Following these revelations, the European Union (EU) launched an EU wide 3-month random sampling DNA testing programme for processed meats (http://webarchive.nationalarchives.gov.uk/+/http://www.food.gov.uk/news-updates/news/2013/5560/eu-sampling) (http://webarchive.nationalarchives.gov.uk/20150624093026/http://www.food.gov.uk/enforcement/monitoring/horse-meat/timeline-horsemeat) (http://www.bbc.co.uk/news/world-21453370) (https://www.gov.uk/government/news/processed-beef-products-and-horse-meat). Extensive testing was conducted throughout the then 27 EU member states with 4144 samples labelled as beef collected (mostly from point of sale outlets, e.g., retailers, quick service restaurants) and tested for horsemeat. Of those samples, 4.66% of samples were said to contain horse DNA (http://europa.eu/rapid/press-release_IP-13-331_en.htm). A further 7951 samples collected from food business operators including producers, processors and distributors were tested; 1.38% of tested samples contained horse DNA (http://europa.eu/rapid/press-release_IP-13-331_en.htm). Testing revealed no traces of horse DNA in meat imported from outside the EU.^3^ From these analyses, it was clear the crisis was not confined to the UK and Ireland but was in fact, an issue of much larger magnitude within the EU; the scandal continued to dominate the British and Irish media for months after^4^ where the consumption of horsemeat was considered morally unacceptable. In other EU member states, horsemeat is regarded as food and is routinely consumed. Substitution or adulteration of meat with another meat species which has religious or cultural connotations e.g., pork is not eaten by Muslim and Jewish communities, could be regarded as a more serious transgression due to the impact on religious or cultural identity and belief systems.

After some initial confusion in the response to the horsemeat crisis, the Food Standards Agency (FSA) took the lead at the country level in the UK, and undertook coordinated testing programmes with local authorities and industry throughout the UK.^5^ Six months after the crisis broke, an independent review into ‘…the integrity and assurance of food supply networks’ was commissioned by the UK government.^6^ The review was carried out by Professor Chris Elliott of Queen’s University, Belfast (and is hereon in referred to as The Elliott Review) and highlighted many recommendations following publication of two reports; the interim report published in December 2013 (https://www.gov.uk/government/uploads/system/uploads/attachment_data/file/264997/pb14089-elliot-review-interim-20131212.pdf) and the final report, published in July 2014 (https://www.gov.uk/government/uploads/system/uploads/attachment_data/file/350726/elliot-review-final-report-july2014.pdf). It was in this review the idea of food crime was first concretely considered as an issue in food supply chains. The review alluded that food fraud becomes food crime when the act of food fraud is no longer a few random acts by so called rogues but a series of organised activities by groups.^2^

In this paper, we aim to identify the measures generated and changes instigated by industry and government in the UK to fulfil the recommendations set out by the Elliott Review to address food fraud issues highlighted by horsegate. Firstly, an overview of the beef supply chain is provided in order to gain an appreciation of the complexity of the beef supply chain, and how this can create vulnerabilities in the chain which can be exploited by food fraudsters intent on committing food crime.

## Background–The UK beef supply chain

**Fig. 1:**
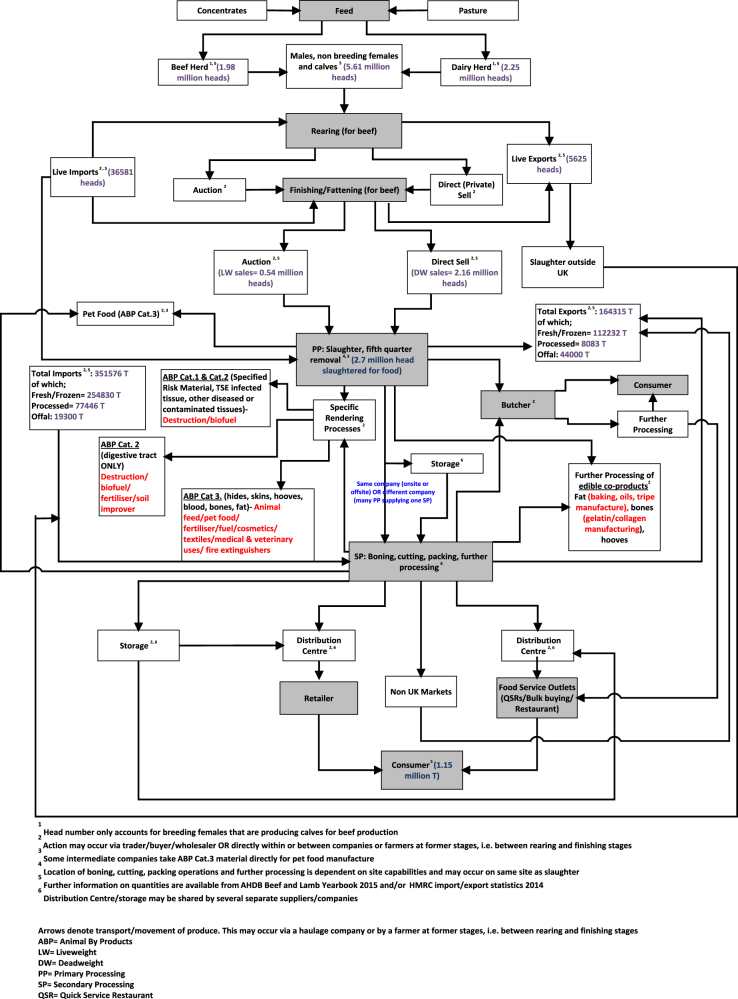
The UK Beef Supply Chain

The beef supply chain is complex with import and export channels at several stages in the chain. Figure 1 illustrates the typical steps involved in producing and transporting beef products through the supply chain to the final consumer. The steps involved at processing and importing/exporting stages, as well as the reliance on storage facilities and the number of actions that can occur directly or via a trader or agent illustrate the particular intricate nature of the beef chain. In some cases, multiple traders can be involved in sourcing and supplying meat and when there are more steps involved greater management of the process is required. The external markets (importing and exporting) play a vital role in the sufficiency and flexibility of the UK beef supply chain.

With beef production susceptible to seasonal changes, poor weather, disease outbreaks, crop failures and therefore feed shortages, the importance of diverse external markets for securing supply should not be underestimated.^7,8^ The illustration by the Guardian Newspaper, provides a simplified version of events for the Irish and French companies connections to horsemeat, (available at: http://www.theguardian.com/uk/graphic/2013/feb/15/horsemeat-scandal-food-safety1) exemplifying the complex nature of their beef supply chains and the multiple entities which can be involved in trading meat. The complex nature and subsequent vulnerabilities of international agri-food supply chains has been evaluated previously in literature.^9^ To meet the requirements of UK consumers, e.g., preferences for certain cuts, sourcing outside domestic supply chains is required in order to secure supply but within the EU single market where trading occurs freely, it can be more challenging to monitor and manage supply chains and consequentially, can expose vulnerabilities resulting in problems such as the horsemeat incident in 2013. The need for companies to ensure supply at a low cost is thought to be a causative factor in the horsemeat scandal. The European Commission stated, ‘The story that horsemeat was being passed off as beef, exposed the complex nature of our globalised food supply chain…It demonstrated that fraudsters were taking advantages of weaknesses in the system to the detriment of both legitimate business and consumers.’ (http://europa.eu/rapid/press-release_MEMO-14-113_en.htm). However, it is important to note that additional controls are placed on meat imports from outside the EU and as such, at the time, horsemeat was not detected in third country consignments. The Elliott Review^6^ affirms the consequences of such complex systems stating, ‘The more complex the supply chains the greater degree of vulnerabilities…’ illustrating the difficulties in managing such systems. This paradox between ensuring supply but also integrity exemplifies the importance of ensuring that the stakeholders are aware of the complexity of their food supply chains and acknowledge, assess and mitigate against risks involved with these chains.

## Results and discussion

The Elliott Review found that while the UK food industry has made overwhelming progress in ensuring that our food is safe to eat, the focus has not been on preventing and protecting against food crime.^4^ As a result, intelligence gathering, horizon scanning and regimented testing were not key elements in the food industry’s risk assessments in the prevention and detection of food crime.^6^ The final report recommended eight pillars of food integrity: consumers first, zero tolerance, intelligence gathering, laboratory services, audit, government support, leadership and crisis management, and were aimed at numerous stakeholders including, the food industry themselves (i.e., producers, processors), regulators and enforcement bodies (government).^2^ The progress made by industry and government in the last 4 years with regard to each of the eight pillar recommendations is detailed below.

### Pillar one—consumer first

In the weeks following the horsemeat incident, consumer trust in beef products decreased to an all-time low (http://www.tifsip.org/european_commission_announces_actions_achieved_following_horsemeat_scandal.html?RequestId=419cdf1b). Many media and research institutions including 'Which?' (http://www.which.co.uk/news/2013/03/horsemeat-scandal-dents-trust-in-food-industry-313016/), 'The Consumer Council'^10^ and 'Kantar Worldpanel' (http://uk.kantar.com/consumer/shoppers/horsemeat-scandal-consumer-behaviour-and-trust/) reported decreases in UK consumer trust in processed meat products and in food retail outlets themselves. Kantar Worldpanel reported a 43% and 13% drop in frozen burger and frozen-ready meals sales, respectively, in the 4 weeks leading up to 17th February 2013 compared to the same period in 2012 (http://www.kantarworldpanel.com/global/News/Grocery-Market-Share-UK-First-Data-Since-Horsemeat-Scandal). Consumers shopping habits changed as a result of horsegate^11^ with a consumer survey revealing 7% of consumers stopped purchasing meat altogether (http://www.economist.com/news/britain/21572230-what-horse-shy-consumers-are-eating-instead-and-winner) and 65% of consumers trusted food labels less as a result of the incident (http://www.reuters.com/article/us-horsemeat-britain-survey-idUSBRE91H0GP20130218). A study into consumer confidence post horsegate, found that consumers expressed a sense of betrayal and concern over the complexity of supply chains.^4^ A Consumer Council report published in July 2013 reported that almost half of (47%) consumers saw food retailers in a less favourable light in the months following the incident^10^ and a 24% decrease overall in consumer trust in the food industry was reported in March 2013 (http://www.which.co.uk/news/2013/03/horsemeat-scandal-dents-trust-in-food-industry-313016/).

The Elliott Review^2^ recommended consumer needs be of the highest priority for industry in order to ensure consumer confidence in the food they purchase. It was recommended that committing food crime should be made as difficult as possible through the prevention of contamination, adulteration and false claims. Which? had consumers interests at the forefront when they began their campaign entitled ‘Stop Food Fraud’ calling on government, the FSA and local authorities to help stop food fraud (http://www.which.co.uk/campaigns/meat-takeaways-horsemeat/). This campaign received 37,000 consumer signatures and is regarded as one of the integral initiatives that helped rebuild consumer trust in the food industry following horsegate.^12^

Since horsegate, Professor Elliott^12^ believes food retailers’ attitudes have changed drastically, with transparency now a key trend. For example, Tesco provide information online on their meat product testing regime and is widely regarded as a mechanism for trust building among consumers (http://www.thegrocer.co.uk/buying-and-supplying/consumers-trust-meat-in-supermarkets-more-than-restaurants-claims-survey/517134.article) (https://www.tescoplc.com/tesco-and-society/further-information-and-disclosure/testing-regime-update/). Following the campaigns and initiatives put in place by the industry, an improvement in consumer trust levels in the meat industry and supermarkets was reported with consumers trust levels returning close to pre horsegate levels (http://www.thegrocer.co.uk/buying-and-supplying/consumers-trust-meat-in-supermarkets-more-than-restaurants-claims-survey/517134.article).^13^ It is believed these campaigns and initiatives taken to put consumers first are claimed to have helped the industry regain consumer trust (http://www.ibtimes.co.uk/man-centre-2013-horse-meat-scandal-arrested-masterminding-new-large-scale-operation-1616003).^12^

### Pillar two—zero tolerance

The Elliott Review^2^ recommended the food industry adopt a zero tolerance to food fraud and industry were encouraged to question whether some procurement deals were ‘too good to be true’, i.e., procurement of raw material that is suspiciously inexpensive in relation to anticipated cost of production, to ensure due diligence in procuring safe and genuine food from reputable sources. Further, food crime/fraud and mitigation strategies were to be considered as part of risk assessment procedures and whistleblowing/reporting of food fraud and crime was strongly encouraged. A whistleblowing hotline has been subsequently set up by the National Food Crime Unit (NFCU), (see pillar 7). Additionally, it was suggested that industry should be rewarded for responsible procurement and sourcing practices and the review encouraged industry to carry out sampling and testing within their supply chains.^2^

Previously within the meat industry, ‘a little bit of cheating’ would have been considered acceptable within the moral boundaries of ‘normal practice’.^12^ For example, horsegate can be seen as a consequence of this ‘little bit of cheating’ where Polish beef was procured, packaged and labelled as British beef. While the food company believed they were operating inside the moral boundaries of ‘normal practice’, the consequences of these practices came when horsemeat was found within meat procured from Poland and sold as British beef. It is a belief that the industries are now more aware that they are being monitored extensively by their customers through the rigorous testing programmes implemented since horsegate and as a result attitudes to cheating have changed.^12^ However, a recent online publication suggests illegitimate actors (fraudsters) are continuing to mastermind and facilitate food fraud (https://www.food.gov.uk/news-updates/news/2016/15226/food-crime-confidential-launch). Despite this, it is believed there is much greater awareness of food fraud and a realisation among legitimate meat industry actors, such as processors, that shortcuts cannot be taken regardless of downstream pressures.^12^ Genuine food businesses are aware that any deviation from customer specification can have great consequences for their businesses.^12^ While this is thought to be the case within the meat industry, this unfortunately may not be the case in other sectors where shortcuts are still considered within ‘normal practices’.^12^ This illustrates that there is a continued tolerance towards food fraud by some actors and they may not cease until abuses result in injury or death as with the Melamine incident in 2008.

### Pillar three—intelligence gathering

Government and industry focus on intelligence gathering and sharing was the take home message from recommendation three.^2^ In addition, the development of a ‘safe haven’ for industry to collect and disclose information and intelligence (whistleblowing) without fear was recommended. Protection of whistle-blowers from retaliation has also been highlighted as a necessity by the United States Food Safety Modernisation Act (https://www.fda.gov/Food/GuidanceRegulation/FSMA/ucm247548.htm). Previously within the food industry technical counterparts from different companies informally discussed suspicious supplier behaviours or practices, but this information was never shared with regulators and governmental departments for fear they would be implicated in the suspicious activity.^12^ Top legal firms in the UK advised their clients not to share information or intelligence with the regulator unless absolutely necessary.^12^

Since horsegate, in the UK, a system has been established to facilitate intelligence gathering and sharing within industry called the Food Industry Intelligence Network (FIIN), which covers all commodities and about 60% of all foods sold in the UK^14^ (http://www.foodmanufacture.co.uk/Food-Safety/Food-fraud-remains-a-threat-to-UK-firms) (https://drive.google.com/drive/folders/0B_9GiFJoLcpwRkhoZmFtcTNKamM). The FIIN network allows companies to share anonymised information and test results via a legal firm which ensures that competitive advantages cannot be gained from one company knowing another’s results. The anonymised information is passed on to the FIIN network for analysis and subsequently shared with member companies. A lot of company resource has been channelled into testing thousands of meat products in light of horsegate and results indicate there to be no detectable fraudulent activity in meat tested at the current time^12^ but this does not suggest food fraud is gone for good. Professor Elliott explains there is a distinct need for product testing to be dynamic to ensure other commodities are examined sufficiently. Despite the significant steps the industry has taken in sharing information and intelligence with each other, information is still not shared with the regulator or government departments as recommended by The Elliott Review, due to issues surrounding confidentiality, competition and fear of implication.^12^ Due to the government’s commitment to a high level of transparency with the public, food businesses have concerns regarding governments (FSA) intentions in sharing of highly sensitive company information such as company name. Food businesses want assurances that personal company information will not be shared publically.^12^ As this written guarantee has not yet been provided, the FIIN network information remains unshared with regulators and government departments. Interestingly, Food Standards Scotland (FSS) has agreed to provide this written guarantee and thus FIIN will share their information with FSS but not the FSA at the present time.^12^ The effect this may have on a potential shift in the FSA’s position remains to be seen.

While not solely a UK initiative, The Food Fraud Network (FNN) is another intelligence gathering initiative established post horsegate. The FNN set up in 2013 and comprising of 28 national food fraud contact points in a European regulatory sharing network^15,16^ (http://www.foodqualitynews.com/Industry-news/EU-improves-cooperation-between-national-authorities) (http://ec.europa.eu/food/safety/official_controls/food_fraud/index_en.htm), working closely with INTERpol and Europol on Operation Opson, targeting organised food crime networks (http://www.octf.gov.uk/OCTF/media/OCTF/images/publications/Government%20Reports/OPSON-Booklet-FINAL-electronic-version.pdf?ext=.pdf). However, this information is not shared with any other party (including industry). Intelligence gathering and sharing was recommended and intended to be a joint activity between regulators and industry. However, this appears not to be the case as industry and regulators are actively gathering and sharing information and intelligence within their respective groups but not with each other.

### Pillar four—laboratory services

The horsemeat scandal revealed that laboratory services for food fraud testing were not up to the required standard. The quality of testing was unknown and the number of laboratories that could provide such services was decreasing due to a downturn in government spending.^2^ In light of this, the Elliott Review^2^ recommended access to resilient and sustainable laboratory services with standardised and validated testing methods be implemented as a priority. It urged the government to facilitate the development of surveillance programmes (intelligence gathering, horizon scanning) and targeted sampling programmes based on intelligence gained.

Professor Elliott revealed that, in the last 4 years, there has been a substantial increase in the number of companies now providing meat testing services such as DNA testing, driven by the market demand and evidenced by a simple google search (https://www.google.co.uk/webhp?sourceid=chrome-instant&ion=1&espv=2&ie=UTF-8#q=meat+authenticity+testing+laboratories). While this is a positive outcome and meat products may be tested adequately, Professor Elliott is concerned that wider food surveillance infrastructures on other products and commodities such as herbs and spices is lacking.

### Pillar five–audit

The Elliott Review^2^ highlighted food audits as too food safety focussed and food fraud awareness within auditing to be poor. Consequently, the review recommended developing a modular approach to auditing underpinned by both food safety and integrity standards. The industry has taken this on board and taken several steps to address the recommendation on a wider industry level. The existing British Retail Consortium (BRC) Food Safety Standard (adopted by manufactures as a pre-requisite for customer supply) has been adapted (Issue 7) to include new mandatory clauses concerning vulnerability assessment^17^ (http://www.brcglobalstandards.com/Manufacturers/Food/FoodIssue7.aspx#.V_txI48rK70). Food manufacturing sites are expected to carry out frequent risk assessments of their products by completing vulnerability assessments on raw materials procured directly (from manufacturer) or indirectly (via agent/broker) and establishing mitigating strategies to reduce any identifiable risk.^17^ Additionally, where raw materials are procured via an agent/broker, sites must understand and assess the manufacturer’s suitability as a supplier.^17^ It is important to note that these new clauses apply to all food sectors and not just the meat sector.

The Elliott Review^2^ identified traders and brokers as an area of vulnerability in the chain and several initiatives have been adopted by industry to address this. The BRC Standard for Agents and Brokers was developed to allow reputable agents and brokers to be certified against a standard which ensures they have processes in place to manage their supply systems, with a particular focus on food fraud prevention.^17^ BRC Global Standards state that UK food retailers are actively encouraging their agents and brokers who supply retailer Own Label produce to become certified against this standard.^17^ The BRC Standard for Agents and Brokers was implemented on a macro industry level and applicable to all agents and brokers (not just meat agents and brokers) and does not take into account the specific nature of the meat industry.^18^ The IMTA Good Trading Practice Guide to food fraud prevention in 2015 is tailored specifically to meat traders and brokers and sought to ‘identify practical steps which companies could take to strength their resilience to fraud threats’ and is available to the International Meat Traders Association (IMTA) members and non-members, on request.^18^ In addition to the guide, IMTA have introduced a ‘meat scam tracker’ where members can report suspect scams across the industry.^18^ Following active engagement with the NFCU, (see pillar 7), IMTA members are seen to be proactively and regularly sharing intelligence with IMTA which is subsequently passed onto the FSA.^18^ Since the horsemeat scandal, IMTA have worked to ensure better information sharing with government and acknowledge the efforts of their members to become more food fraud aware via the implementation of preventative measure in their businesses.^18^

In conjunction with the food industry and the British Meat Processors Association, BRC Global Standards have also developed a voluntary bolt on module to the Food Safety Standard specifically for meat companies called Meat Supply Chain Assurance, which goes back to the point of slaughter.^17^ This modules enables the ability ‘…to demonstrate to customers an increased transparency of their meat supply chains…’ (http://www.brcglobalstandards.com/Manufacturers/Food/VoluntaryModules.aspx#.V8lG1U0rIdU), and is designed to enable meat companies to demonstrate increased traceability and visibility in their supply chain and how they manage species–species contamination.^18^ The Meat Supply Chain Assurance bolt on is believed to have removed some of the additional audits triggered in the immediate aftermath of horsegate.^17^

The Elliott Review^2^ recommended regulators and industry work together to develop an appropriate auditing training platform for food fraud detection. While this has resulted in a market opportunity for auditing bodies/companies, it is still in the early stages of inception.^12^ Training of auditors to be forensically food fraud aware is fraught with difficulties as training materials and methods cannot enter the public domain as this would create an awareness of the auditing scope and criteria and thus facilitating fraudulent companies and individuals to commit further food crime/fraud.^12^

The review recommended that the number of audits be decreased through using a risk assessed approach, i.e., giving credit where credit is due, but the quality of these audits should be improved to ensure food fraud is an integral part.^2^ Contrary to other beliefs, Professor Elliott believes the number of audits are thought to have actually increased in light of horsegate, with individual customers continuing to carry out their own audits.^12^ Auditing is a business in itself, where substantial amount of money is generated, and could be regarded as a conflict of interest to rationalising and reducing the number of audits.^12^ However, there have been significant steps taken by food retailers to move to an unannounced platform as recommended by The Elliott Review; 99.5% of Asda audits and more than 50% of Tesco audits are now unannounced, while M&S has created a new unannounced audit platform specifically looking at Food Integrity.^12^

### Pillar six—government support

Ensuring a joint and inter-disciplinary approach to fighting food fraud between high level officials in FSA, Department for Environment, Food and Rural Affairs (DEFRA) and Department of Health (DH) was recommended by The Elliott Review.^2^ In light of this, the Cross-Government Group on Food Integrity and Food Crime has been established and is chaired by the DEFRA Minister for Food and Farming, George Eustice and attended by ministers from the DH (Public Health), Home Office (Organised Crime), Business, Innovation & Skills (Consumer Affairs) and the Chair of the FSA.^19^ The high level official group meet bi-annually with senior officials in these departments providing support through regular meetings. It is the regular collaboration and communication between senior officials, outside of high level meetings, that creates connectivity between departments.^12^

### Pillar seven—leadership

The nature and magnitude of the horsegate incident was something not previously encountered by the regulatory authorities, and consequently meat fraud had been overlooked previously.^5^ On the other hand, there is widespread awareness of fraud in the olive oil and fish industries. The uniqueness of the situation meant that in the initial days and weeks after the incident broke, there was a lack of clarity in who was to respond and lead the incident—the FSA or DEFRA?.^5^ This was reaffirmed in the Elliott Review where it was identified that there was no one body dedicated to fighting and preventing food crime/fraud in the UK and as a result, the introduction of a NFCU was recommended.^2^ The Elliott Review investigated food crime investigation infrastructures in other countries, namely the Netherlands, Denmark, France and Germany and concluded the UK system should be modelled on a system similar to that employed by the Dutch which has full police powers and has been established for over 60 years.^2^

The NFCU, set up in December 2014 was placed within the FSA and is headed by a former senior intelligence officer with experience in several law enforcement agencies including the Serious Fraud Office and National Crime Agency.^20^ The role of the NFCU is to ‘give greater focus to enforcement against food fraud in government by analysing intelligence, initiating investigations and liaising with other criminal and regulatory enforcement agencies’ and leads the response to food crime in England, Wales and Northern Ireland.^20^ In June 2016, the NFCU launched Food Crime Confidential; a whistleblowing hotline where food crime can be reported safely and anonymously (https://www.food.gov.uk/news-updates/news/2016/15226/food-crime-confidential-launch). While significant steps have been taken to implement the NFCU, it is regarded as significantly under resourced with a £900,000 annual budget (as opposed to £2–4 million per year as recommended by The Elliott Review), to carry out any meaningful activities.^12^ The new Chair of the FSA has recently commissioned a review of the NFCU to determine its future direction.^12^

### Pillar eight—crisis management

The Elliott Review recommended that effective mechanisms needed to be implemented in order to sufficiently deal with serious food safety and/or food crime incidents in the future.^2^ The review stressed the importance of defining roles and responsibilities in the FSA before another food safety and/or food crime incident surfaces. While there hasn’t been another major red meat-related incident since horsegate, there has been other major food fraud incidents uncovered in other sectors including in the herbs and spices,^21^ honey (http://ec.europa.eu/food/safety/official_controls/food_fraud/honey/index_en.htm) and fish (http://ec.europa.eu/food/safety/official_contr) industries. A study carried out by the Institute for Global Food Security at Queen’s University Belfast found 24% of oregano samples purchased from UK retailers and online sources had been adulterated with other non-oregano ingredients.^21^ Professor Elliott believes there has been a significant learning in dealing with major food incidents and government is now much better equipped to deal with major food fraud incidents, compared to when horsegate surfaced in 2013.

It is believed there is a much greater understanding among industry and government of how food fraud incidents like horsegate can occur in these complex supply chains. While significant improvements in the understanding of fraud and the application of fraud prevention have been made in industry and government, it is important to note that ‘fraud hasn’t gone away, [and that] fraud [type] just changes’.^12^ This illustrates that the fight against food fraud/crime is a continuous process as fraudsters continue to evolve and find innovative ways to infiltrate supply chains as long as human greed exists. It is therefore important to ensure mitigation and detection strategies are at least equally innovative and evolutionary.

By mapping the UK beef supply chain, this paper has provided context as to how the nature of complex and convoluted supply chains can create vulnerabilities open to exploitation by opportunistic food fraudsters, particularly within the EU. The Elliott Review’s eight pillars of integrity recommended measures to help improve the integrity of food supply systems. Significant steps have been made by both industry and government to implement some of the recommendations in the UK. Industry attitudes to ‘a little bit of cheating’ have changed substantially. Testing and surveillance systems that have been integrated into normal industry practice and the government are more prepared for future incidents through the establishment of the NFCU and the defining of roles and responsibilities. Horsegate substantially raised the profile of food fraud and crime occurrence within supply chains and despite improvements to date, further collaboration between industry and government is required in order to align fully with the recommendations set out in The Elliott Review. Ensuring the sharing of intelligence across industry and government and adequate resource allocation are particularly important in the fight against food fraud. Lessons learnt in the UK and Ireland in relation to horsemeat and the subsequent implemented recommendations and initiatives from The Elliott Review are not only applicable to UK and Irish contexts. They are also relevant to other jurisdictions to enable the implementation of safeguards in preventing food fraud and crime in their own country.

## Methods

Using personal communications with key representatives of relevant industry bodies, BRC Global Standards, the IMTA and Professor Chris Elliott), this paper aims to provide an overview of the actions and changes put in place by industry and government in line with the recommendations set out in the Elliott Review. Ethical approval was gained from the Research Ethics Committee at Queen’s University Belfast and the research was completed in line with guidance under the Declaration of Helsinki. Prior consent from interviewees was gained and interviews were carried out between August and September 2016.

### Data availability

The authors declare that the data supporting the findings of this study are available within the paper.
